# Nitrate, Ascorbic Acid, Mineral and Antioxidant Activities of *Cosmos caudatus* in Response to Organic and Mineral-Based Fertilizer Rates

**DOI:** 10.3390/molecules17077843

**Published:** 2012-06-28

**Authors:** Siti Aishah Hassan, Salumiah Mijin, Umi Kalsom Yusoff, Phebe Ding, Puteri Edaroyati Megat Wahab

**Affiliations:** 1 Department of Crop Science, Universiti Putra Malaysia, Serdang Selangor 43400, Malaysia; 2 Department of Biology, Universiti Putra Malaysia, Serdang Selangor 43400, Malaysia

**Keywords:** fertilizer source, ascorbic acid, antioxidants, nitrate content

## Abstract

The source and quantity of nutrients available to plants can affect the quality of leafy herbs. A study was conducted to compare quality of *Cosmos caudatus* in response to rates of organic and mineral-based fertilizers. Organic based fertilizer GOBI (8% N:8% P_2_O_5_:8% K_2_O) and inorganic fertilizer (15% N, 15% P_2_O_5_, 15% K_2_O) were evaluated based on N element rates at 0, 30, 60, 90, 120 kg h^−1^. Application of organic based fertilizer reduced nitrate, improved vitamin C, antioxidant activity as well as nitrogen and calcium nutrients content. Antioxidant activity and chlorophyll content were significantly higher with increased fertilizer application. Fertilization appeared to enhance vitamin C content, however for the maximum ascorbic acid content, regardless of fertilizer sources, plants did not require high amounts of fertilizer.

## 1. Introduction

Plants that are naturally high in antioxidant properties will supply the body with essential dietary antioxidant components to supplement their natural defense system. However, management practices such as source and rate of fertilizer used can affect plant nutrient composition and quality [1,2]. In green vegetables, besides vegetative growth impact, fertilizer has been reported to influence vitamin and antioxidant activities [3]. Fertilizer effects on antioxidant activity in medicinal plants has also been reported [4,5,6]. In common agricultural practices, inorganic fertilizer is used to maximize the production of foliage for leafy herbs. However, environmental considerations and consumer awareness about clean healthy produce had limited the quantity and sources of nutrients that can be applied. Health conscious consumers are interested in the nutritional composition of herbs, preferring those with minimal chemical residues and herbs that produced through environmentally friendly agricultural practices. Hence substituting chemicals with organic fertilizers is one of the vital production techniques to meet the demand. Compost has been widely used as a source of nutrients and reported to influence phytochemical content in the products [1,7]. Compared to mineral fertilizers, organics resulted in higher tannin and pectin content in date fruits [8], and higher total antioxidant capacity in cabbage [9] and herbs [6,10]. Fruits, vegetables and grains from organic crops were reported to contain significantly more vitamin C, iron, magnesium, and phosphorus and significantly less nitrates than conventional crops [11].

Although fertilizer is a greatly needed input in plant growth, excess fertilizer use can result in serious damage to the soil and ecosystem, ground or surface water contamination, and quality of the produce. The quantity of fertilizer used in nutrient management has important effects on the quality and sustainable production of herbs. Antioxidant levels in *Brassica rapa* seemed to decrease as the fertilizer rate increased, especially under conventional fertilization [12]. Lila [13] and Carsky and Lwuafor [14] have reported that a plant will contain higher levels of phytochemicals and antioxidant compounds if it has experienced some stress due lower rates of fertilization during its development. The effects of nitrogen rate on antioxidant properties of herbs had been reported. Increasing nitrogen fertilizer increased the concentration of lutein and carotene of parsley [15] and phenolic compounds and carotenoids in lavender [16], whereas in *Chrysanthemum morifolium* heavy nitrogen fertilization was reported to decrease flavonoids and antioxidant activity of flowers [17]. Quality parameters like dry matter, specific gravity, starch contents, vitamin-C and ash contents were also affected with P and K fertilization [18]. A proper potassium level is needed for good vitamin C levels. Increased application of fertilizers resulted in an increased in vegetative growth and yield of *Aronia melanocarpa* whereas the content of anthocyanins and total acidity decreased [19].

While fertilizer impact on vegetative growth is well documented, the effect of fertilizer rate and sources on phytonutritional quality is still contradictory. Many studies have demonstrated inconsistent differences in the nutritional quality of conventionally and organically produced vegetables with the exception of nitrate and ascorbic acid [20]. The young shoots of *Cosmos caudate*, as a leafy herb, are freshly consumed as a good source of natural antioxidants, minerals and vitamins B1, B2 and C [21]. It is recommended in traditional medicine for blood circulation and healthy bones [22]. Being a less known herb, not much is known about its antioxidant activity in relation to fertilizer application. The objective of this study was to determine the effect of fertilizer source and application rates on phytonutritional quality of *Cosmos caudatus*.

## 2. Results and Discussion

There are many environmental and cultivation factors that influence the nutritional composition of produce, and may ultimately play a greater role in food quality. Cultivation practices likely to affect food quality include humus management techniques such as green manuring and composting, variety, irrigation and fertilization. Besides for plant growth and development, research is uncovering the fact that availability of plant nutrients and water can be important factors in determining secondary metabolite synthesis within plants [23,24,25]. Reports indicate that high nutrient availability leads to an increase in plant growth and development, but a decrease in allocation of resources favours production of secondary metabolites. Sources of fertilizer have been also documented to influence the nutritional quality of vegetables and fruits [11,26].

**Figure 1:**
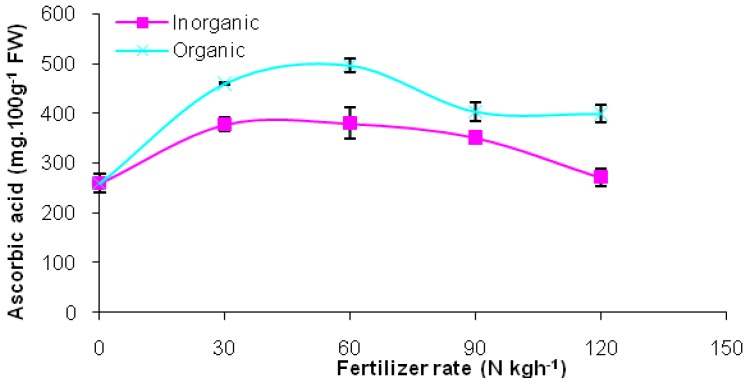
Ascorbic acid content in leaf tissue of *Cosmos caudatus* in response to fertilizer rates and sources.

The nutritional value of *Cosmos caudatus* depended on the experimental factors. Ascorbic acid content significantly varied with the rates and sources of nutrients (Table 1). Organic treatments significantly improved vitamin C content compared to the synthetic fertilizer. This may be due to differences in composition between organic and inorganic fertilizers and their effects on soil ecology and plant metabolism. Similar findings on ascorbic acid content trends in organically produced fruits and vegetables were also reported [11,27]. For the maximum ascorbic acid content, regardless of fertilizer sources, plants did not require high amounts of fertilizer. Quadratic responses were shown in relation to fertilizer rates. In both cases, within the tested rates, this crop probably only requires a fertilizer rate ranging between 30 to 60 kg h^−1^ of N for optimum ascorbic acid production (Figure 1). As such, consumers would still get healthy fresh herbs without causing contamination to environment. Reduction in ascorbic acid content in leaf tissue resulting from higher fertilizer application rates was also reported in other crops [28].

**Table 1:** Ascorbic acid, nitrate and chlorophyll concentration in leaf tissue of *Cosmos caudatus* in response to fertilizer rates and sources.

Treatment	Ascorbic acid (mg·100 g^−1^ FW)	Nitrate (ppm)	Chlorophyll (mg/cm^2^)
Fertilizer sources Inorganic	328.43a	261.3a	30.90a
Organic	411.90b	193.3b	30.13a
Fertilizer rates (N kg h^−1^)			
0	259.7c	115.0b	24.45c
30	419.8a	241.7a	29.25b
60	438.8a	243.3a	31.10b
90	378.3b	244.2a	35.83a
120	354.3b	292.5a	31.95ab
Source × Rate	ns	ns	ns

ns, Non significant or significant at *p* ≤ 0.05, respectively. Means followed by the same letter are not significantly different by LSD test (*p* ≤ 0.05).

Nutrient compositions in shoot tissues can also be used to evaluate the quality of fresh leafy herbs. It is well known that leafy vegetables are one of the important sources of mineral nutrients for human daily intake. The amount of nutrient supply in growth media is reflected in the quantity of nutrients being absorbed by the plant. Mineral content in leaf tissues was influenced by fertilizer rates. All elements analyzed were significantly increased with fertilizer application. In the experiment compound fertilizer was used and each fertilizer has similar percentages of the elements N, P and K. Nitrogen was used as a basis to determine the quantity of fertilizer applied. Among elements, nitrogen concentration was the most affected. Nitrogen content was significantly increased with increasing quantity of fertilizer applied (Table 2). Phosphorous and calcium only significantly differed with control. Organic fertilizer had a higher N concentration. This is probably due to the slow-release nutrients of organic fertilizer and their beneficial effects on soil improvement. Compost and manure improve the physical, chemical and biological properties of soil [29] and help to overcome loss of nutrients through leaching processes. This is particularly important in the production of healthy herbal crops that are to be used as natural remedies. Nitrogen is the building block for amino acids and protein synthesis as well as chlorophyll composition. Improvement of N concentration resulted from higher fertilizer application had led to significant improvement in chlorophyll contents. Chlorophyll will be reflected in leaf colour. Greenness is one of the quality criteria that consumers prefer for fresh leafy herbs.

**Table 2:** Nutrient contents in leaf tissue of *Cosmos caudatus* in response to fertilizer rates and sources.

Treatment	Nutrient content (%/g DW)				
	N	P	K	Ca	Mg
Fertilizer sources Inorganic	3.22b	0.44a	2.22a	0.31b	0.13a
Organic	3.49a	0.41a	2.18a	0.34a	0.14a
Fertilizer rates (N·kg h^−1^)					
0	2.73d	0.35b	2.04b	0.38b	0.18b
30	3.44c	0.43a	2.24ab	0.52a	0.19b
60	3.63b	0.45a	2.23ab	0.52a	0.21a
90	3.59bc	0.45a	2.20ab	0.53a	0.22a
120	3.88a	0.46a	2.31a	0.55a	0.21a
Source × Rate	*	ns	ns	ns	ns

ns, *, Non significant or significant at *p* ≤ 0.05, respectively. Means followed by the same letter are not significantly different by LSD test (*p* ≤ 0.05).

**Figure 2:**
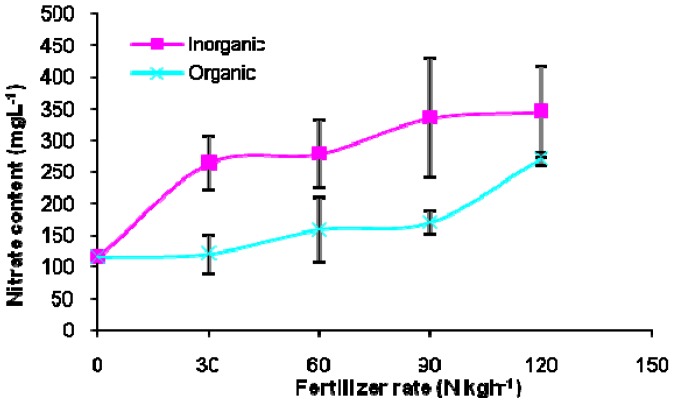
Nitrate content in leaf tissue of *Cosmos caudatus* in response to different nutrient sources and rates.

Nitrogen in plant cells can be in the form of nitrate and ammonium. Nitrate has been postulated to produce detrimental effect to human health [30]. Symptoms of nitrate toxicity include headaches, syncope, vertigo and cutaneous discoloration that manifests in the fingers or lips. The young shoots of *C. caudatus* are normally consumed fresh as a salad. However, reports have indicated that young plants in the vegetative stage generally contain more nitrate than mature plants, even of the same species. This is especially true of young pasture plants that have been liberally fertilized with nitrogen [31]. In endive, nitrate content was increased from 1,806 to 2,015 mg·100 g^−1^ of fresh weight at a N rate of 90 and 135 kg h^−1^, respectively [32]. In our results, nitrate level increased with higher levels of fertilizer applied (Table 1). The concentration of nitrate of inorganic fertilized plants was significantly higher than in organic crops. When organic based fertilizer was used, the nitrate concentration was lower even at higher fertilizer levels (Figure 2). Fertilizers used in organic farms contain nitrogen bound to organic material from which it is slowly released [33]. Readily soluble chemical fertilizers which are absorbed rapidly into the plant tend to lead to higher nitrate/nitrite levels and may result in formation of nitrosamines which have been associated with chronic diseases such as leukaemia and gastrointestinal cancers [34]. Nitrate is harmful to humans hence organic fertilizer should be wisely used for fresh herb production to minimize the the negative effects of fertilizer application. Results evidently indicate that organic-based fertilizer plays an important role in the move towards more natural and healthier food production methods.

**Figure 3:**
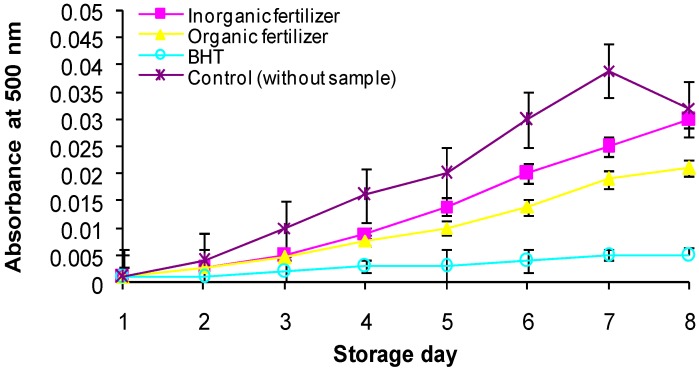
Effect of fertilizer sources on antioxidant activity of *Cosmos caudatus* using FTC method.

**Figure 4:**
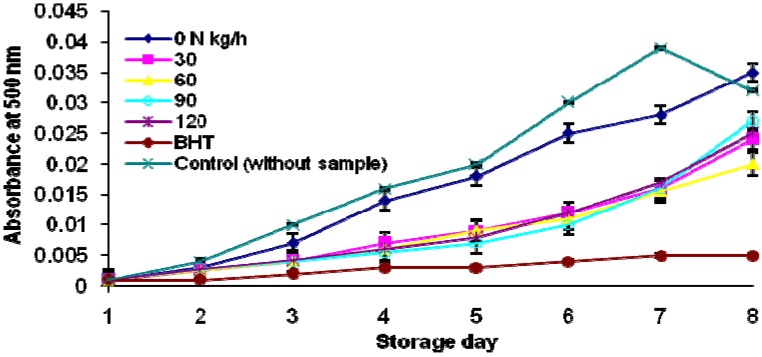
Effect of fertilizer rates on antioxidant activity of *Cosmos caudatus* using FTC method.

The antioxidant activity of compounds derived from natural resources has attracted the attention of those who are concerned about their healthy diet. The antioxidant activity of ulam raja was tested using the ferric thiocyanate (FTC) and thiobarbituric acid (TBA) methods. In the FTC method, the amount of peroxide in the initial stages of lipid oxidation was measured every 24 h, over a period of 7 days (the absorbance of the positive control reached a maximum on the sixth day). In parallel with the ascorbic acid finding, a higher antioxidant activity of organic compared to non-organic fertilized *C. caudatus* was also detected (Figure 3). Results from the FTC method indicated that antioxidant activity in *C. caudatus* was influenced by fertilization rates (Figure 4). Low absorbance values indicate high level of antioxidant activity. Irrespective of fertilizer sources, fertilized plants exhibited higher antioxidant activity than those without fertilization. The activity however was not significantly different amongst fertilized plants. 

**Figure 5:**
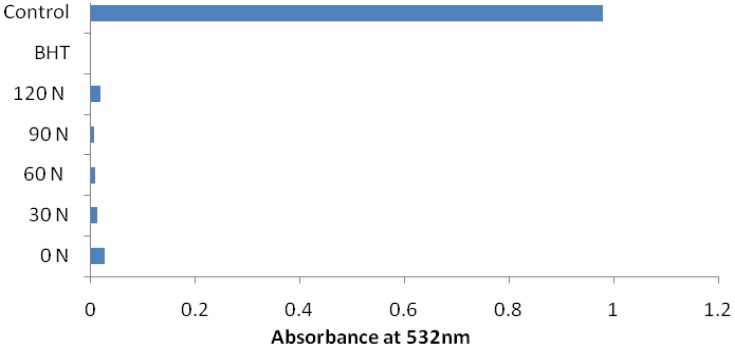
Effect of fertilizer rates on antioxidant activity of *Cosmos caudatus* determined using TBA method. The TBA values were measured on the 8th day after storage; control (without sample); 0, 30, 60, 90, 120 N kg h^−1^.

The TBA method measured the degradation of peroxide to lower molecular weight compounds during oxidation. The absorbance data using the TBA test at 8 days of storage is shown in Figure 5. The absorbance values for 30 to 90 kg h^−1^ N treatment were considerably lower than that of plants without fertilization, indicating their stronger antioxidative properties. There was no significant interaction difference between fertilizer sources and nitrogen rates.

The positive effect of fertilizer on nutritional quality of *C. caudatus* may be due to an overall promoting effect on general metabolic activities. This could be explained by differences in mineral composition in the fertilizer used, as well as improvement in soil properties. Application of compost not only provides nutrients, but also prevents soil erosion, improves soil physical, chemical and biological properties [35]. Organic fertilizer can be considered as a complete fertilizer as it contains major and minor elements, whereas the chemical fertilizer used in this study supplies mainly the three major elements (15% N, 15% P_2_O_5_ and 15% K_2_O). Plants grown under organic agricultural conditions are reported to have higher micronutrient content in more cases than conventionally grown plants [36]. Considering the fact that some of chemical reactions in cells involve minor elements, either directly or indirectly [37], this could explain why organic fertilized plants exhibited higher antioxidant activity as well as ascorbic acid content. 

## 3. Experimental

The factorial experiment was conducted under a rain shelter at University Putra Malaysia (02°N 59.476' 101°E 2.867', 51 m altitude). The climatic conditions recorded under the rain shelter were 30 °C mean temperature, 90% humidity and 60% light at 12 a.m. Seeds were germinated in seedling trays and transplanted into polybags (14 × 18 cm) filled with a mixture of top soil, organic matter and river sand (3: 2: 1 v/v). The media had a pH value of 6.15. Coffee based organic fertilizer GOBI^®^ (8% N: 8% P_2_O_5_: 8% K_2_O) and inorganic fertilizer (15% N, 15% P_2_O_5_, 15% K_2_O) were used as sources of nutrients and evaluated based on N element rates at 0, 30, 60, 90, 120 kg h^−1^. Plants were irrigated manually. Inorganic fertilizer was split equally in three applications and applied at 2, 4 and 6 weeks after transplanting whereas for organic fertilizer, 50% of the total was applied at transplanting and the remaining fertilizer 3 weeks after. Treatments were arranged in randomized complete block design with three replicates. Shoots were harvested at weeks eight after transplanting. The newly expanded leaves were used to determine nutrients, chlorophyll, nitrate and ascorbic acid contents and antioxidant activity. The leaves were oven-dried, ground and subjected to H_2_SO_4_-H_2_O_2_ digestion for quantitative nutrient analysis [38]. Total N and P were measured using auto analyzer (System 4 Chemlab) whilst an atomic absorption spectrophotometer (Perkin Elmer Model 310) was used for K, Ca and Mg. The chlorophyll content was measured following the method suggested in [39]. Three discs of 1 cm^2^ of fresh leaves were sampled by using cork borer. Samples were placed in 80% acetone (20 mL) with pH of 7.8 and kept in the dark at room temperature for seven days to ensure maximum chlorophyll content was extracted from the tissues. The chlorophyll concentration in extract was measured using a spectrophotometer (U-2001, Hitachi Instruments Inc., Tokyo, Japan) at 663.6 and 646.6 nm for chlorophyll a and b, respectively. Leaf samples were also collected and kept in a refrigerator prior to nitrate and ascorbic acid analysis. The fresh leaves were cut to small pieces and squeezed in a stainless steel press to obtain sap. Sap is then used to measure nitrate concentration using a Cardy nitrate meter (Spectrum Technologies, Inc. Springfield, IL, USA) following [40]. Ascorbic acid was measured by titration method [41]. Five grams of fresh leaves were blended to pulp in 3.0% metaphosphoric acid (HPO_3_, 45.0 mL), filtered and the extract was gradually titrated with dye solution (2,6-dichlorophenol indophenol) until the colour changed to pink. Ascorbic acid content was then calculated based on the following formula: ascorbic acid (mg)/100 g = (titre × dye factor × volume made up × 100)/(aliquot of extract × volume of sample).

For extraction of antioxidant compounds, the leaves were air dried at room temperature for 48 h. Three g of air dried ground leaf tissue was soaked in 98% methanol (50 mL) for 48 h. The sample was stirred every 18 h and the final extract was filtered. The extract was vacuum dried on a rotary evaporator at 40 °C and the filtrate (final concentration 0.02% w/v) obtained was kept at 4 °C before being subjected to the bioassays [42]. The antioxidant activity of plant extracts were assessed by ferric thiocyanate (FTC) and thiobarbituric acid (TBA) methods. The FTC assay was carried out as described by Kikuzaki and Nakatani [42] with slight modification. Four mg of sample was placed in screw-cap vials and added with 99.5% ethanol (4 mL), linoleic acid (2.51% linoleic acid prepared in 99.5% ethanol, 4.1 mL), 0.02 M phosphate buffer (pH 7.0, 8.0 mL) and distilled water (3.9 mL). Butylated hydroxytoluene (BHT) was used as a standard antioxidant while the other bottle without sample was used as a control. Then 75% (v/v) aqueous ethanol (9.7 mL), followed by 3% aqueous ammonium thiocyanate (0.1 mL) and 0.02 M ferrous chloride in 3.5% hydrochloric acid (0.1 mL) was added. Three minutes after the addition of ferrous chloride to the mixture, the absorbance was measured at 500 nm using the U-2001 spectrophotometer. The measurement was taken every 24 h until absorbance of the control reached its maximum value. The TBA test was also conducted according to the method of [42]. The same samples prepared for the FTC method were used. Two mL of the sample solution was added with 20% aqueous tricloroacetic acid (1 mL) and 0.67% aqueous thiobarbituric acid solution (2 mL). The mixture was placed in a boiling water bath for 10 min. After cooling, it was centrifuged at 3,000 rpm for 20 min. Antioxidant activity was measured at 532 nm (measured absorbance of the supernatant at the final day of the FTC assay) using the U-2001 spectrophotometer. All data were analyzed using analysis of variance (SAS Institute, Cary, NC, USA) and subjected to LSD test for means separation.

## 4. Conclusions

Increasing fertilizer application affected the phytonutritional content of *C. caudatus*. The results showed that organically fertilized shoots have significantly higher ascorbic acid and antioxidant activity and less nitrate levels than the corresponding non-organic fertilized *C. caudatus*. Considering nitrate is harmful, whereas mineral and vitamin contents as well as antioxidant activity are priorities in fresh herbs, organically grown produce may benefit human health better than the corresponding conventionally grown produce. This finding is consistent with a general improvement in the antioxidant system developed by the plant in organic vegetables and fruits [11].
